# Conservative Treatment and Surgical Indication of Cervical Ossification of the Posterior Longitudinal Ligament

**DOI:** 10.3390/jcm12175719

**Published:** 2023-09-01

**Authors:** Takeo Furuya, Kenichiro Sakai, Toshitaka Yoshii, Masaaki Machino

**Affiliations:** 1 Department of Orthopaedic Surgery, Chiba University Graduate School of Medicine, 1-8-1 Inohana, Chuo Ward, Chiba-shi 260-8670, Japan; 2 Department of Orthopedic Surgery, Saiseikai Kawaguchi General Hospital, 5-11-5 Nishikawaguchi, Kawaguchi-shi 332-8558, Japan; kenitiro1122@gmail.com; 3 Department of Orthopedic Surgery, Tokyo Medical and Dental University, 1-5-45 Yushima, Bunkyo Ward, Tokyo 113-8519, Japan; yoshii.orth@tmd.ac.jp; 4 Department of Orthopedic Surgery, Meijo Hospital, 1-3-1 Sannnomaru, Naka Ward, Nagoya-shi 460-0001, Japan; masaaki_machino_5445_2@yahoo.co.jp

Ossification of the posterior longitudinal ligament (OPLL) sometimes causes severe myelopathy and requires surgical treatment. The presence of ossification of the ligaments is a risk factor for the incidence of spinal cord injury [1]. However, there are a certain number of patients with large ossification of the posterior longitudinal ligaments who remain asymptomatic or experience only mild numbness for a long period of time.

**1.**  **Conservative Therapy for Cervical OPLL**

Conservative therapy for cervical radicular pain and axial pain is performed using various medications, including anti-inflammatory analgesics, muscle relaxants, vitamin B12, neuropathic pain medications, steroids, and weak opioids. There remains no effective treatment myelopathy, despite the high level of research in this field. In clinical practice, physiotherapy and orthosis are often used in combination with medications. In mild cases, occasionally, patients may spontaneously recover provided that they rest. Ethane-1-hydroxy-1,10diphosphonate (EHDP, Daidronel^®^) is reported to be effective in preventing the progression of ossification [2]. EHDP inhibits calcification and bone resorption and is used as a treatment for heterotopic ossification after spinal cord injury and hip arthroplasty.

**2.**  **The Natural Course of Cervical OPLL and the Appropriate Timing of Surgery**

Many asymptomatic patients with OPLL do not develop neurological symptoms. One review, published in 2011, indicates that the possibility of symptom deterioration is low in the absence of myelopathy at first visit. On the other hand, if myelopathy is already present, the risk of symptom deterioration is high and surgical treatment may be effective [3]. A three-dimensional CT study of the progression of OPLL showed that the average rate of ossification increase was 4.1%/year [4]. It is generally recognized that there is no correlation between ossification progression and symptoms. Factors that may influence neurological symptoms include ossification morphology, residual effective spinal canal diameter (ossification occupancy ratio) at the maximum compression level [5] and dynamic factors at the relevant level [6,7]. The deterioration of neurological symptoms is often segmental-type or mixed-type in terms of OPLL morphology. All patients with an interspinal canal diameter of 6 mm or less present with myelopathy, and static factors may be a factor in the development of myelopathy; in patients with a spinal canal diameter of 6 mm or more, dynamic factors, rather than static factors, are more likely to be involved in the development of myelopathy.

Neurologically, patients who maintain a JOA score of 15 or higher can essentially be treated conservatively; many spine surgeons consider a JOA score of around 12 to 13 to be the cut-off point indicating the option of surgery.

As the myelopathy progresses, patients complain of gait disturbance and clumsy hand syndrome. Considering the timing of surgery based on symptoms, it is common to consider surgery when patients begin to find daily activities difficult due to myelopathy. If the patient does not experience spastic gaits, clumsy hand syndrome, or bladder and bowel disturbance, and the only symptom is numbness, the option of surgery should be discussed in detail with the patient. The option of surgery for neck pain should also be carefully considered. As mentioned, OPLL is a risk factor for spinal cord injury. However, prophylactic surgery in patients with no symptoms or small symptoms is should often be considered with great caution because spinal surgery always carries a certain risk of surgical complications.

As an indicator of surgery based on imaging findings, the risk of future symptom progression at an occupancy rate of 40% or greater, or a spinal canal diameter of 8 mm or less, has been reported [8]. In addition, based on the results of a study of patients undergoing long-term conservative treatment, patients with an occupancy rate of 60% or more are at higher risk of developing symptoms and should be followed up more carefully [5].
